# Proline-Rich Tyrosine Kinase 2 (Pyk2) Promotes Cell Motility of Hepatocellular Carcinoma through Induction of Epithelial to Mesenchymal Transition

**DOI:** 10.1371/journal.pone.0018878

**Published:** 2011-04-20

**Authors:** Chris K. Sun, Kevin T. Ng, Zophia X. Lim, Qiao Cheng, Chung Mau Lo, Ronnie T. Poon, Kwan Man, Nathalie Wong, Sheung Tat Fan

**Affiliations:** 1 Department of Surgery, LKS Faculty of Medicine, Centre for Cancer Research, The University of Hong Kong, Pokfulam, Hong Kong, China; 2 Department of Anatomical and Cellular Pathology, The Chinese University of Hong Kong, Shatin, Hong Kong; Virginia Commonwealth University, United States of America

## Abstract

**Aims:**

Proline-rich tyrosine kinase 2 (Pyk2), a non-receptor tyrosine kinase of the focal adhesion kinase (FAK) family, is up-regulated in more than 60% of the tumors of hepatocellular carcinoma (HCC) patients. Forced overexpression of Pyk2 can promote the proliferation and invasion of HCC cells. In this study, we aimed to explore the underlying molecular mechanism of Pyk2-mediated cell migration of HCC cells.

**Methodology/Principal Findings:**

We demonstrated that Pyk2 transformed the epithelial HCC cell line Hep3B into a mesenchymal phenotype via the induction of epithelial to mesenchymal transition (EMT), signified by the up-regulation of membrane ruffle formation, activation of Rac/Rho GTPases, down-regulation of epithelial genes E-cadherin and cytokeratin as well as promotion of cell motility in presence of lysophosphatidic acid (LPA). Suppression of Pyk2 by overexpression of dominant negative PRNK domain in the metastatic HCC cell line MHCC97L transformed its fibroblastoid phenotype to an epithelial phenotype with up-regulation of epithelial genes, down-regulation of mesenchymal genes N-cadherin and STAT5b, and reduction of LPA-induced membrane ruffle formation and cell motility. Moreover, overexpression of Pyk2 in Hep3B cells promoted the phosphorylation and localization of mesenchymal gene Hic-5 onto cell membrane while suppression of Pyk2 in MHCC97L cells attenuated its phosphorylation and localization.

**Conclusion:**

These data provided new evidence of the underlying mechanism of Pyk2 in controlling cell motility of HCC cells through regulation of genes associated with EMT.

## Introduction

Hepatocellular carcinoma (HCC) is the primary malignancy of the liver. It is fifth in popularity and third in cancer-related deaths worldwide [1]. Prognosis and treatment of HCC remains unsatisfactory due to tumor recurrence, metastasis of the primary tumor and poor therapeutic response to chemotherapy and radiotherapy [2], [3]. Metastasis is not only a complex process but also the major cause of cancer-related deaths [4]. Transformation of cells to a fibroblastic phenotype is important for the cancer cells to successfully metastasize [5]. Several lines of evidences suggested that the induction of epithelial to mesenchymal transition (EMT) plays an important role in cancer cell transformation [6], [7]. It contributes significantly to metazoan embryogenesis and pathogenesis such as tissue fibrosis and cancer progression [8]. On the other hand, the process of mesenchymal to epithelial transition (MET) may promote the growth of the metastatic cancer cells in secondary sites [9]. The critical hallmarks of EMT include the down-regulation of E-cadherin which is considered to be a tumor suppressor gene [10], activation of Rho small GTPases such as Rac1/RhoA which may increase cell motility by up-regulating actin turnover and formation of focal adhesion [11], cytoskeletal rearrangement and nuclear translocation of several transcription factors such as Snail and Twist [12], [13]. Understanding the mechanism of HCC cell migration and metastasis may have great value to develop effective diagnostic and therapeutic strategies for treatment of HCC patients.

Proline-rich tyrosine kinase 2 (Pyk2) is a non-receptor tyrosine kinase of the focal adhesion kinase (FAK) family. Our previous study had shown that up-regulation of Pyk2 in tumor tissues of HCC patients is significantly associated to poor prognosis [14]. Moreover, forced overexpression of Pyk2 in HCC cells promotes cell proliferation, invasion and migration via the activation of the c-Src and ERK/MAPK pathways which can be attenuated by forced overexpression of its C-terminal non-kinase region (PRNK)[15]. Furthermore, Pyk2 up-regulates the formation of lamellipodia and actin stress fiber polymerization of HCC cells [15]. However, the underlying mechanism of Pyk2 on regulation of cell transformation and motility of HCC cells is poorly understood. Recently, some of the signaling molecules associated with Pyk2 (Hic-5 and STAT5b) have been reported to promote EMT [16], [17]. Therefore, it is valuable to investigate the effect of Pyk2 on regulating these molecular in the process of cell transformation of HCC cells.

Hydrogen peroxide inducible clone-5 (Hic-5) is a 55 kDa protein that serves as an adaptor protein in focal adhesion and possesses the ability to translocate to the nucleus, where it acts as a transcription factor [18]. It maintains the general structure of paxillin with 4 N-terminal LD motifs and 4 C-terminal LIM domains [19], [20]. The LIM domain of Hic-5 is able to bind with DNA fragments in a zinc-finger-dependent manner *in vitro*, suggesting its possible role as a transcription factor [21]. Pyk2 can physically interact with Hic-5 and subsequently phosphorylate Hic-5 [22]. It has been reported that activated Pyk2 phosphorylates Hic-5 at tyrosine residue 60 resulting in up-regulation of LPA-mediated cell migration and the induction of EMT through a RhoA/ROCK-dependent pathway [17], [23], [24]. So far the interaction between Pyk2 and Hic-5 in HCC is still unknown.

STAT5b belongs to the Signal Transducers and Activators of Transcription (STAT) family which are activated by cytokines and transcription factors via its dimerization and nuclear translocation. They are involved in a variety of cell processes including cell proliferation, cells survival and differentiation [25], [26], [27]. STAT5b has been reported to promote EMT in HCC by the transformation of HCC cells into an aggressive phenotype. It has been shown that the activation of STAT5b is closely associated with the X protein of the Hepatitis B virus [16].

In this study, we hypothesize that Pyk2 transformed HCC cells to a fibroblastoid phenotype through regulation of genes associated with EMT. To validate the hypothesis, Hep3B cells were stably overexpressed with full length Pyk2 and MHCC97L cells were stably transfected with PRNK to suppress the Pyk2 activation. The gene expression profiles associated with EMT (Hic-5, STAT5b, E-cadherin, Twist, N-cadherin, fibronectin and Rho GTP-binding proteins) were investigated.

## Materials and Methods

### Reagents, plasmids and antibodies

Platelet derived growth factor-BB (PDGF-BB) was purchased from Calbiochem (Darmstadt, Germany). Lysophosphatidic acid (LPA) was purchased from Sigma (St. Louis, MO, USA). Plasmids pCDNA3-Pyk2 and pCDNA3-PRNK were gifts from Dr. Joseph Loftus, Mayo Clinic Scottsdale, USA. pCDNA 3.1 (+) vector was purchased from Invitrogen (Carlsbad, CA). Anti-E-cadherin, anti-N-cadherin, anti-Twist and Anti-phosphotyrosine monoclonal antibodies were purchased from Cell Signaling (Danvers, MA, USA). Monoclonal antibodies against cytokeratin (AE1/AE3) was purchased from DAKO (Glostrup, Denmark). Anti-STAT5b and anti-fibronectin antibodies were purchased from Santa Cruz (Santa Cruz, CA, USA). Anti-Hic-5 and anti-Pyk2 monoclonal antibodies were purchased from BD Transduction Laboratory (San Jose, CA, USA). Alexa fluor 488 goat anti-rabbit IgG and goat anti-mouse IgG were purchased from Molecular Probes (Carlsbad, CA, USA).

### Cell culture, transfection and stable cell lines

Human HCC cell line Hep3B was purchased from the American Type Culture Collection (Manassas, VA, USA) and grown in DMEM medium containing 10% FBS, 2 mM L-glutamine, and 100 units/ml streptomycin (Life technologies, Carlsbad, CA, USA). Human metastatic HCC cell line MHCC97L was a gift from Prof. Z.Y. Tang, Fudan University, Shanghai, China. Stable transfectants MHCC97L-vector and MHCC97L-PRNK has been reported previously. Hep3B cells were stably transfected with full length Pyk2 or PCDNA 3.1 (+) empty vector and maintained in DMEM medium supplemented with 300 µg/ml G418 as described previously [15]. The expressions of Pyk2 in the stable clones were confirmed by Western blotting. One clone with the highest expression of Pyk2 was selected for further study.

### Scanning electron microscopy (SEM)

Cells were seeded in 13-mm glass dishes and maintained in serum-free DMEM medium supplemented with 10% FBS and antibiotics G 418 (0.6 mg/ml for Hep3B cells and 0.2 mg/ml for MHCC97L cells). To study the effects of Rho small GTPases activation on cell transformation, the cells were further treated with PDGF-BB (10 ng/ml) or LPA (1 µg/ml) for 10 minutes before cell fixation. Cells were fixed with 2.5% glutaraldehyde in 0.1 M sodium cacodylate-HCL buffer, pH 7.4, quenched with 0.1 M sucrose/cacodylate solution and washed in cacodylate buffer. The samples were then post-fixed with 1% OsO_4_ in cacodylate buffer. After a cacodylate buffer wash, samples were dehydrated through a graded series of ethanol washes, followed by critical point drying using a Bal-Tec CPD 030 (Bal-Tec AG, Liechtenstein). The samples were sputter-coated with a thin layer of gold (Bal-Tec SCD005 Sputter Coater) and visualized using a Leica Cambridge Stereoscan 440 SEM at an accelerating voltage of 12 kV. Each experiment was repeated for 3 times.

### Activation assays for Rac1 and RhoA

Rac and RhoA activation assays were purchased from Cytoskeleton (Denver, CO, USA). Hep3B transfectants were serum-starved overnight prior to experiment. Cells were treated with PDGF-BB (10 ng/ml) or LPA (1 µg/ml) for 5 minutes and were then lysed with lysis buffer. Rac1 and RhoA pull-down assays were performed according to manufacturer's protocol.

### Migration assay

Cells were trypsinized, counted and resuspended in serum free DMEM medium. Around 50,000 cells were seeded on the upper side of the migration chamber (BD, San Jose, CA, USA) with the lower chamber supplemented with serum free DMEM medium or with LPA (1 µg/ml). After 36 hrs cells that had penetrated through the chamber were fixed and counted.

### Immunofluorescence staining

The protocol for immunofluorescent staining has been reported previously [15]. Briefly, cells were fixed and stained with anti-E-cadherin, anti-Hic-5 and anti-STAT5b antibodies. The cells were then labeled with alexa fluor 488 goat anti-rabbit IgG or goat anti-mouse IgG and counter-stained with DAPI at 37^o^C.

### Immunoprecipitation and Western blotting

Immunoprecipitation was performed on whole-cell lysates using antibodies against Hic-5 and phosphotyrosine. The cells were serum starved for 24 hours before stimulation with LPA for 20 minutes. Cell lysates were then incubated with anti-Hic-5 antibody for 4 hours at 4^o^C. Immunoprecipitates were washed twice in ice-cold lysis buffer. Immunoblotting was carried out as previously reported [15].

### Statistics and data analyses

All data were expressed as mean ± SD. Difference between groups were calculated by one-way ANOVA analysis. A *p*<0.05 was considered statistically significant. Unless stated, all experiments were repeated three times. Calculations were performed by using the SPSS computer software version 12 (SPSS, Chicago, IL).

## Results

### Pyk2 promoted formation of membrane ruffles

**Figure 1:**
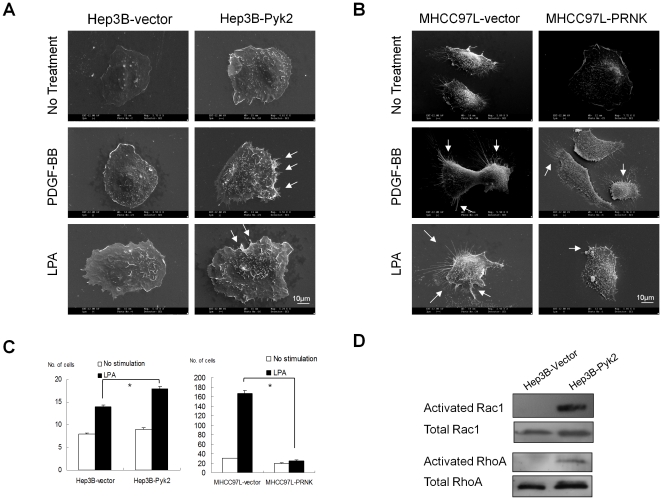
Pyk2 promoted cell motility of HCC cells. (A) Overexpression of Pyk2 in Hep3B cells enhanced the formation of membrane ruffles while (B) suppression of Pyk2 in MHCC97L cells reduced the formation of membrane ruffles under the stimulation of PDGF and LPA. White arrows indicate the presence of membrane ruffles. (C) Effects of Pyk2 on cell migration of Hep3B and MHCC97L cells in presence of LPA. *, P<0.05. (D) The effect of over-expression of Pyk2 on the activation of Rac1 and RhoA in Hep3B cells by Rac1 and RhoA pull-down assays. Total Rac1 and RhoA were used as loading controls.

Forced overexpression of Pyk2 in the non-metastatic HCC cell line Hep3B (Hep3B-Pyk2 cells) enhanced the formation of lamellipodia and membrane ruffles upon stimulation by PDGF-BB or LPA compared to control Hep3B-vector cells (Fig. 1A). Suppression of Pyk2, by overexpression of PRNK domain, in the metastatic MHCC97L cells (MHCC97L-PRNK cells) reduced the formation of membrane ruffles, as compared to the MHCC97L-vector control (Fig. 1B) in presence of PDGF-BB or LPA. Migration assay demonstrated that Hep3B-Pyk2 exhibited a significant increase of LPA-induced migration than Hep3B-vector (*p*<0.05, Fig. 1C). Suppression of Pyk2 activation by PRNK significantly down-regulated the cell migration in MHCC97L-PRNK cells as compared to the vector control (*p*<0.05, Fig. 1C).

To elucidate the contributing mechanisms to the increase of membrane ruffles formation in Hep3B-Pyk2 cells, Rac1 and RhoA pull-down assays were performed to study the effects of Pyk2 on LPA-induced activation of Rac1 and RhoA. Significant up-regulation of activated forms and baseline levels of Rac1 and RhoA were found in Hep3B-Pyk2 cells compared to Hep3B-vector cells (Fig. 1D). For MHCC97L cells, no activated Rac1 and RhoA was detected in their cell lysates due to low baseline levels of Rac1 and RhoA (data not shown).

### Pyk2 down-regulated the expression of E-cadherin and cytokeratin

**Figure 2:**
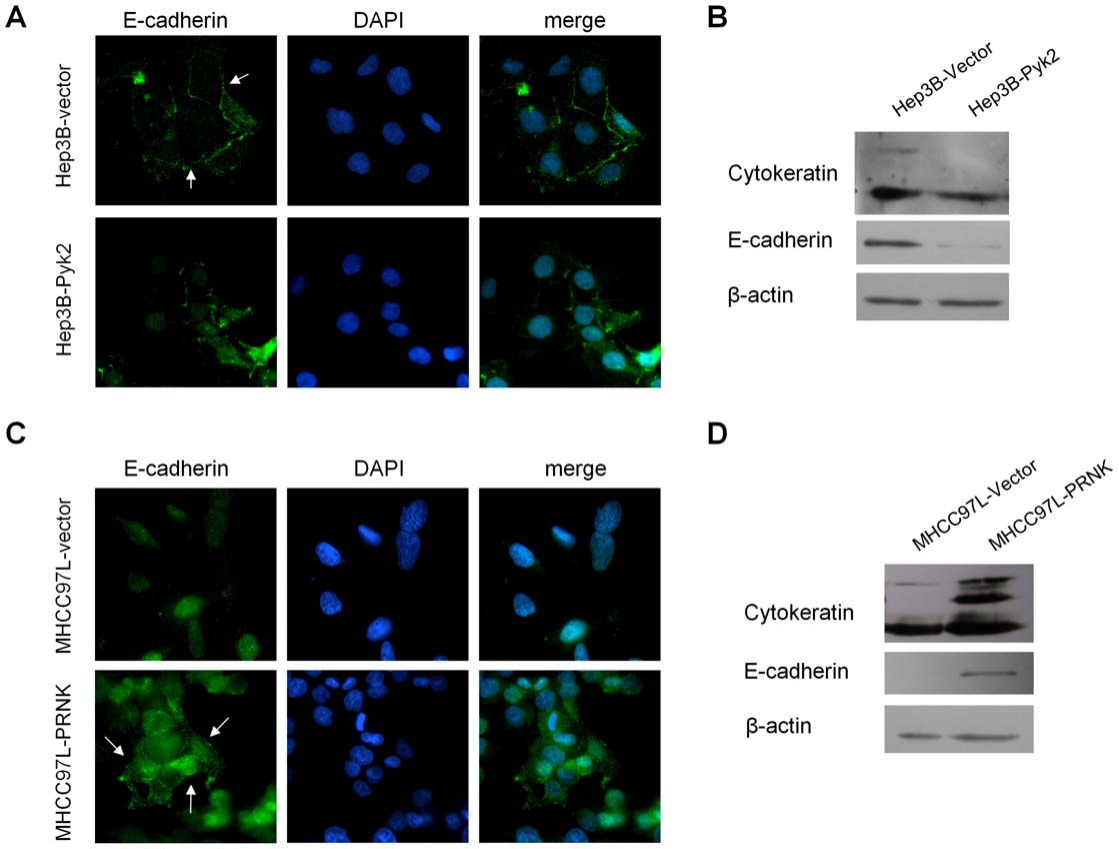
Effects of Pyk2 on epithelial genes cytokeratin and E-cadherin. (A) Effects of Pyk2 over-expression on the localization of E-cadherin in Hep3B cells. Positive staining of E-cadherin (green, white arrow) was present in the cytoplasm of the transfectants. (B) Over-expression of Pyk2 down-regulated the expression of cytokeratin and E-cadherin in Hep3B cells as shown by Western Blotting. (C) Forced expression of PRNK in MHCC97L cells restored the expression of E-cadherin (green, white arrow) on the cell membrane and cytoplasm. (D) The expression of E-cadherin and cytokeratin was increased upon suppression of Pyk2 in MHCC97L cells.

The effects of overexpression of Pyk2 on the regulation of epithelial genes E-cadherin and cytokeratin were investigated. Result from immunofluorescent staining showed that Hep3B-Pyk2 cells had lower level of E-cadherin expression and localization on the cell membrane as compared to the Hep3B-vector cells (Fig. 2A). Weak positive staining (green) was present in the cytoplasm of Hep3B-Pyk2 cells, indicating the deactivation of E-cadherin. Western blot analysis showed that the expression levels of E-cadherin and cytokeratin were lower in Hep3B-Pyk2 cells compared with that in Hep3B-vector cells (Fig. 2B). In MHCC97L-vector cells with high baseline level of Pyk2, E-cadherin expression was almost absent in the cytoplasm of the cells (Fig. 2C). Transfection of PRNK in MHCC97L cells significantly restored the expression of E-cadherin at cytoplasm and on cell membranes (green, Fig. 2C). Western blot analysis showed that suppression of Pyk2 activation by PRNK restored the expression of E-cadherin and cytokeratin (Fig. 2D) in MHCC97L-PRNK cells. These results confirmed the role of Pyk2 on down-regulation of epithelial genes E-cadherin and cytokeratin.

### Pyk2 activated Hic-5 and its focal adhesion localization

**Figure 3:**
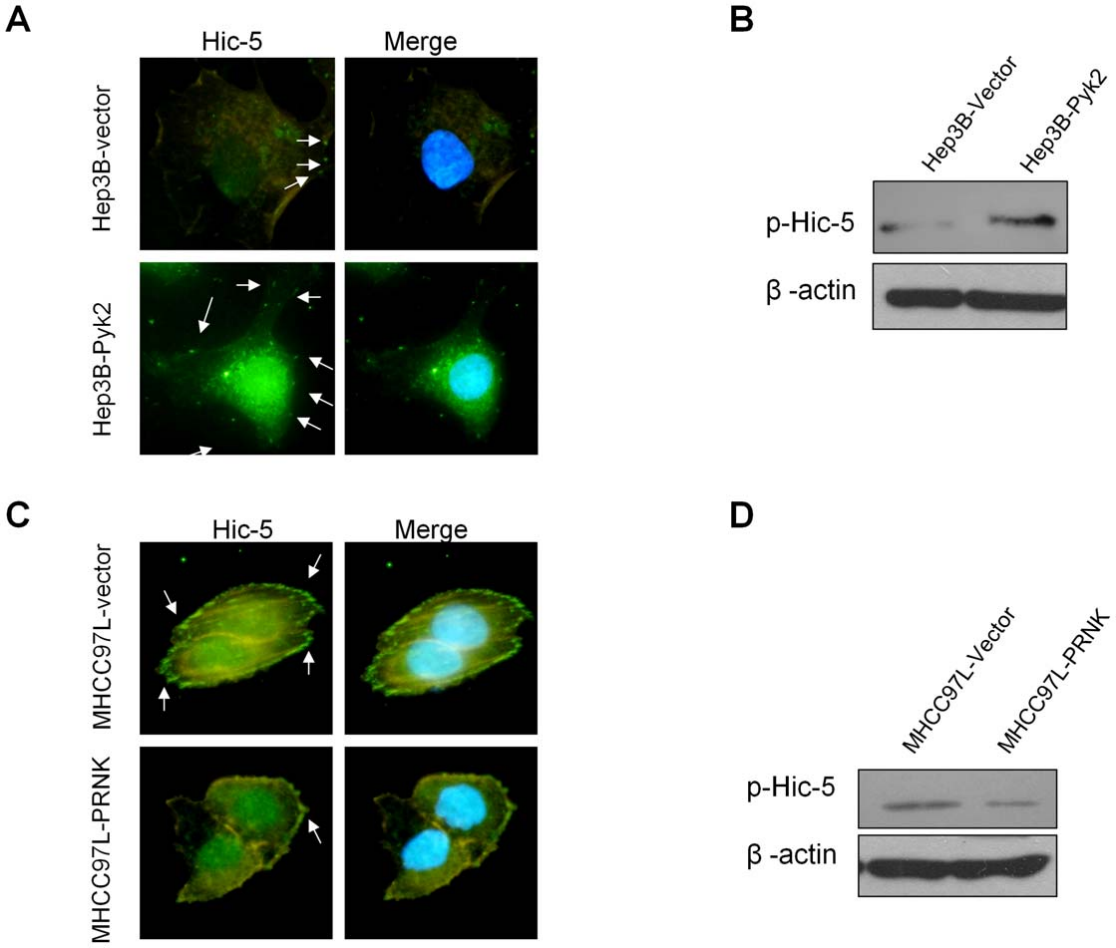
Effects of Pyk2 on the phosphorylation and activation of Hic-5. (A) Pyk2 over-expression promoted the membrane localization of Hic-5. Positive staining of Hic-5 (green, white arrow) was up-regulated in Pyk2 transfected Hep3B cells. (B) Effect of Pyk2 over-expression on the phosphorylation of Hic-5 in Hep3B cells. (C) Suppression of Pyk2 by PRNK down-regulated the localization of Hic-5 (green, white arrow) on cell membrane and (D) level of activated Hic-5 in MHCC97L cells.

Inmmunofluorescent staining demonstrated that Hic-5 was predominantly present in the cytoplasm and the cell membrane of the Hep3B-vector cells in a dispersed pattern (Fig. 3A). Overexpression of Pyk2 promoted the membrane localization of Hic-5 in Hep3B-Pyk2 cells. Interestingly, more positive Hic-5 signals were present on the membrane and the peri-nuclear region in the Hep3B-Pyk2 cells (Fig. 3A). To further confirm the up-regulation of phosphorylated Hic-5 by Pyk2 overexpression, immunoprecipitation was performed to determine the presence of phosphorylated Hic-5 in cultured cells. The level of phosphorylated Hic-5 was up-regulated in Hep3B-Pyk2 cells, as compared to the Hep3B-vector control (Fig. 3B). In MHCC97L cells, Hic-5 was frequently localized on the membrane region as shown by the strong green positive staining by IF (Fig. 3C). Suppression of Pyk2 activation in MHCC97L-PRNK cells significantly down-regulated the membrane localization of Hic-5 (Fig. 3C). Moreover, Western blot result showed that suppression of Pyk2 resulted in the down-regulation of phosphorylated Hic-5 in MHCC97L-PRNK cells (Fig. 3D). These results confirmed the positive correlation between the expression of Pyk2 and the degree of phosphorylation and membrane localization of mesenchymal gene Hic-5 inside the cell.

### PRNK down-regulated the expression and activation of STAT5b

**Figure 4:**
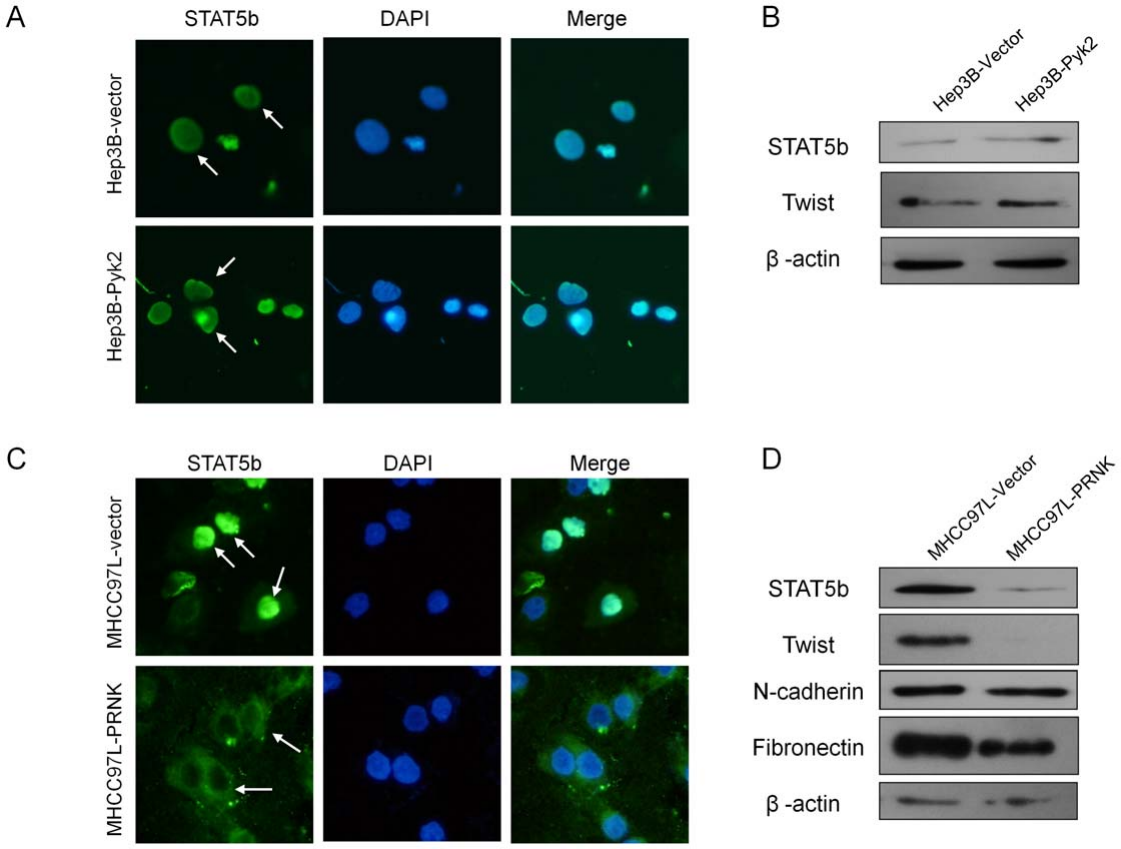
Effects of Pyk2 on the activation of STAT5b. (A) Pyk2 over-expression did not increase activation of STAT5b in Hep3B cells (green, white arrow). (B) Western blot analyses of protein level of phosphorylated STAT5b and Twist between Hep3B-vector and Hep3B-Pyk2 cells. (C) Suppression of Pyk2 down-regulated the nuclear localization of STAT5b (green, white arrow) in MHCC97L cells. (D) Suppression of Pyk2 down-regulated the phosphorylated STAT5b and other mesenchymal genes including Twist, N-cadherin and fibronectin analyzed by Western blot.

In Hep3B-vector cells with low endogenous expression of Pyk2, green positive immunofluorescent staining of STAT5b was localized in the nuclear region of the cells, indicating its activation. Overexpression of Pyk2 did not significantly enhance the activation of STAT5b because of its intrinsic activation in the vector control (Fig. 4A). Western blot analysis showed that overexpression of Pyk2 in Hep3B did not up-regulate the expression of mesenchymal genes including STAT5b and Twist (Fig. 4B). For MHCC97L-vector cells, STAT5b was predominantly localized at the nucleus. Activated dimerized STAT5b, was present in the nucleus (green; Fig. 4C). Suppression of Pyk2 in MHCC97L-PRNK cells resulted in a loss of nuclear staining of STAT5b. Positive staining of STAT5b was observed in the peri-nuclear region and the cytoplasm, but not in the nucleus (Fig. 4C). Western blot analysis confirmed the down-regulation of STAT5b expression in MHCC97L-PRNK cells compared to that in MHCC97L-vector cells (Fig. 4D). In MHCC97L cells, suppression of Pyk2 activation by PRNK resulted in down-regulation of other mesenchymal genes such as Twist, N-cadherin and fibronectin (Fig. 4D).

## Discussion

We previously demonstrated that the focal adhesion localization is a major determinant for Pyk2 to carry out its function in HCC cells [15]. In this study, the role and underlying molecular mechanism of Pyk2 on transformation of HCC cells to undergo EMT were investigated. Forced overexpression of Pyk2 in Hep3B cells transformed the cells from an epithelial phenotype to a migratory phenotype which is commonly acquired by cancer cells to induce EMT leading to the increase of metastatic potential [28]. Enhanced formation of membrane ruffles and migration ability in Pyk2-overexpressing Hep3B cells suggested the promoting effect of Pyk2 on cell motility of HCC cells. Increased activation of Rac1/RhoA in Hep3B-Pyk2 cells indicated that Pyk2 might promote cell motility of HCC cells via regulation of Rac1/RhoA activity. Suppression of Pyk2 in a metastatic HCC cell line MHCC97L showing a repression of EMT and cell migratory ability of the cells also indicated the importance of Pyk2 in regulation of cell motility of HCC cells.

The constitutive expression of E-cadherin in both normal and cancer cells may help to maintain adherence junctions and subsequently decrease the cell's capacity to invade or migrate through the extracellular matrix [29], [30]. E-cadherin is frequently down-regulated during tumor progression. Loss of E-cadherin is associated with increased tumorigenecity and metastasis of cancer cells, providing a close correlation between metastasis and EMT [31]. Down-regulation of E-cadherin is usually coupled with an up-regulation of N-cadherin [32]. In this study, overexpression of Pyk2 in Hep3B cells resulted in a down-regulation of E-cadherin expression while suppression of Pyk2 by PRNK in MHCC97L cells significantly up-regulated the expression of E-cadherin, suggesting the important mechanism of Pyk2 on the regulation of adhesiveness of HCC cell via regulation of E-cadherin expression.

The transcription factor, Hic-5, plays important roles in tumorigenesis and metastasis of human cancers including prostate cancer [33] and breast cancer [34]. Hic-5 is also a crucial EMT regulator of cancer cells [20], [24]. Our results demonstrated that overexpression of Pyk2 in Hep3B cells up-regulated the activation of Hic-5 and its focal adhesion localization. Moreover, suppression of Pyk2 activation in MHCC97L cells down-regulated the membrane localization of Hic-5. Hic-5 is a Pyk2-binding protein which is phosphorylated by Pyk2 at tyrosine residue 60 upon physical interaction [22], [23]. Given the role of Hic-5 on cell transformation as reported by other investigators [17], [24], it may be one of contributing factors to the Pyk2-mediated cell transformation in HCC. STAT5b and Twist have been reported to promote aggressiveness and EMT of cancer cells in HCC [16], [35]. In this study, we demonstrated that Pyk2 also regulated the expression of STAT5b and Twist in HCC cells, suggesting a multiple regulatory roles of Pyk2 on EMT associated genes.

In this study, forced overexpression of Pyk2 in epithelial cancer cell line Hep3B up-regulated the mesenchymal gene expression. However, some of the mesenchymal genes were not up-regulated in this study. This may be explained by the partial mesenchymal characteristics of the Hep3B cells, which resulted in an incomplete induction of EMT by Pyk2. In contrast, suppression of Pyk2 activation in MHCC97L cells resulted in the down-regulation of the expression a series of mesenchymal genes including STAT5b, Twist, N-cadherin and fibroectin. These results showed that Pyk2 regulated the expression of mesenchymal genes and promoted the migratory characteristics of HCC cells which can be attenuated by the forced expression of PRNK to suppress Pyk2 activation. For cells with a mesenchymal phenotype (MHCC97L cells), transfection of PRNK successfully transformed the cells to an epithelial phenotype, with the down-regulation of mesenchymal gene expression and up-regulation of epithelial gene expression. E-cadherin was up-regulated with the down-regulation of N-cadherin. The results showed that suppression of Pyk2 by PRNK transformed the cells from a mesenchymal phenotype, to an epithelial phenotype, by undergoing mesenchymal to epithelial transition (MET). These results confirmed the involvement of Pyk2 in the regulation of EMT of HCC cells.

Both EMT and MET are important processes in cancer progression. The induction of EMT in cancer cells may enhance their cell motility and invasiveness so that to facilitate the development of metastasis [13], [36]. Subsequently, circulating tumor cells must undergo MET to initiate growth in a secondary site [37]. Failure of MET induction in bladder cancer cells resulted in micrometastasis, instead of secondary tumors, as shown by experimental models [9]. Novel therapeutic strategies targeting the MET process could be made to inhibit the development of cancer metastasis by preventing secondary tumor formation. In this study, we had shown that the process of both EMT and MET are regulated by the alternation of Pyk2 activation. Overexpression of Pyk2 in Hep3B cells resulted in the induction of EMT. On the other hand, transfection of PRNK in MHCC97L cells resulted in the induction of MET, suggesting that prevention of focal adhesion by targeting of Pyk2 may transform HCC cells from a mesenchymal phenotype to an epithelial phenotype. However, its underlying mechanism is still unclear and thus considered to be worthwhile for further study. Our previous study also demonstrated that the suppression of Pyk2 by PRNK domain in MHCC97L cells can suppress *in vivo* intrahepatic tumor growth and venous invasion as well as extrahepatic lung metastasis [15]. Together, our data suggested the possible role of Pyk2 in the regulation of EMT, MET and metastasis in HCC cells.

Our study demonstrated the important role of Pyk2 on controlling cell motility of HCC cells through regulation of genes associated with both mesenchymal and epithelial transformations. Targeting of Pyk2 should be a promising therapeutic strategy to reduce HCC metastasis.
